# HIV/AIDS knowledge, attitudes and behaviour of persons with and without disabilities from the Uganda Demographic and Health Survey 2011: Differential access to HIV/AIDS information and services

**DOI:** 10.1371/journal.pone.0174877

**Published:** 2017-04-13

**Authors:** Julie Abimanyi-Ochom, Hasheem Mannan, Nora Ellen Groce, Joanne McVeigh

**Affiliations:** 1 School of Health and Social Development, Deakin University, Melbourne, Victoria, Australia; 2 School of Nursing, Midwifery and Health Systems, Health Sciences Centre, University College Dublin, Dublin, Ireland; 3 Leonard Cheshire Disability and Inclusive Development Centre, Department of Epidemiology and Public Health, University College London, London, United Kingdom; 4 Centre for Global Health and School of Psychology, Trinity College Dublin, Dublin, Ireland; Universita degli Studi di Perugia, ITALY

## Abstract

Uganda is among the first to use the Washington Group Short Set of Questions on Disability to identify persons with disabilities in its Demographic and Health Survey. In this paper, we review the HIV Knowledge, Attitudes and Behaviour component of the 2011 Ugandan Demographic and Health Survey, analysing a series of questions comparing those with and without disabilities in relation to HIV/AIDS knowledge, attitudes and practices. We found comparable levels of knowledge on HIV/AIDS for those with and those without disabilities in relation to HIV transmission during delivery (93.89%, 93.26%) and through breastfeeding (89.91%, 90.63%), which may reflect increased attention to reaching the community of persons with disabilities. However, several gaps in the knowledge base of persons with disabilities stood out, including misconceptions of risk of HIV infection through mosquito bites and caring for a relative with HIV in own household (34.39%, 29.86%; *p*<0.001; 91.53%, 89.00%; *p* = 0.001, respectively). The issue is not just access to appropriate information but also equitable access to HIV/AIDS services and support. Here we found that persons with multiple disabilities were less likely than individuals without disabilities to return to receive results from their most recent HIV test (0.60[0.41–0.87], *p*<0.05). HIV testing means little if people do not return for follow-up to know their HIV status and, if necessary, to be connected to available services and supports. Additional findings of note were that persons with disabilities reported having a first sexual encounter at a slightly younger age than peers without disabilities; and persons with disabilities also reported having a sexually transmitted disease (STD) within the last 12 months at significantly higher rates than peers without disabilities (1.38[1.18–1.63], *p*<0.01), despite reporting comparable knowledge of the need for safer sex practices. This analysis is among the first to use HIV/AIDS-related questions from Demographic Health Surveys to provide information about persons with disabilities in Uganda in comparison to those without disabilities. These findings present a more complex and nuanced understanding of persons with disabilities and HIV/AIDS. If persons with disabilities are becoming sexually active earlier, are more likely to have an STD within the preceding 12 month period and are less likely to receive HIV test results, it is important to understand why. Recommendations are also made for the inclusion of disability measures in Uganda’s AIDS Indicator Survey to provide cyclical and systematic data on disability and HIV/AIDS, including HIV prevalence amongst persons with disabilities.

## Introduction

There is a tribe of Ugandans … whose issues and needs have not been given their due and appropriate attention in the fight. By all indications, persons with disabilities have been forgotten, consciously and unconsciously. They represent “the forgotten tribe”. (Mwesigwa Martin Babu) [1] (p.30)

Uganda has witnessed widespread HIV/AIDS for a quarter of a century [2]. However, while continuing to experience high rates of HIV infection, AIDS mortality, and children orphaned by AIDS, Uganda is regarded as a success story, effectively reducing HIV prevalence from 18% in 1992 to 6% by 2004 [2]. A strengthened HIV response in 2014 resulted in an increase in uptake of HIV prevention, treatment and care services, resulting in continued reduction of the number of new HIV infections and AIDS related deaths [3]. However, the number of persons living with HIV in Uganda continues to increase, due to the on-going spread of HIV, and increased longevity of persons living with HIV [3]. In Uganda, the number of people living with HIV in 2015 was estimated by UNAIDS to be approximately 1,500,000; the prevalence of adults aged between 15 and 49 was approximately 7.1% [4].

HIV/AIDS and disability are closely interwoven [5,6]. Disability, defined by the United Nations Convention on the Rights of Persons with Disabilities (UNCRPD) [7] as resulting “from the interaction between persons with impairments and attitudinal and environmental barriers that hinders their full and effective participation”, is often experienced as both a risk factor for and consequence of HIV/AIDS. The United Nations advises that persons with disabilities are at increased risk of exposure to HIV; furthermore, persons with HIV/AIDS are at risk of developing a disability on a permanent or episodic basis as a consequence of their condition [8]. For example, persons with disabilities are frequently excluded from HIV education, prevention and support services due to misconceptions that they are not sexually active or do not partake in risk behaviours including drug use; healthcare services may be physically inaccessible, lack sign language facilities and other information formats such as Braille, audio or plain language; and when access to medication is limited, persons with disabilities may be treated as low priority for services [8]. Research suggests that persons with disabilities experience a lack of information about HIV/AIDS [2,9,10]. At present, however, relatively little is known about the association between disability and HIV/AIDS, and only limited prevalence data is available for populations with disabilities in sub-Saharan Africa [5,9,11–20].

Accordingly, there is an urgent need for research on disability and HIV/AIDS [12,14,21]. As advocated by Mitra [18], a disability data revolution is needed to enable disability-inclusive development by including disability in data collection and monitoring mechanisms in international development and global health. Data collection is crucial to disability-related planning and programming. As Hanass-Hancock *et al*. [22] have argued, a dearth of research on disability may be a significant contributor to the present invisibility of disability in national HIV and AIDS programmes. Further epidemiological research is required to create a more in-depth understanding of the multifaceted links between disability and HIV to assist decision-makers in prioritising interventions [23]. For example, De Beaudrap and colleagues [23] outline a protocol for a population-based survey combining qualitative and quantitative data collection methods including the Washington Group questionnaire [24], designed to understand the vulnerability of persons with disabilities to HIV in Cameroon.

In a 2013 review of the intersection between HIV/AIDS and disability, Groce *et al*. [15] asserted that household surveys need to include questions to identify persons with disabilities. The authors suggested a need for standardized questions to allow comparable data collection and analyses, such as the Washington Group Short Set of Questions on Disability (Washington Group Short Set) [15,25]. Uganda is among the first to use the Washington Group Short Set of Questions on Disability to identify persons with disabilities in its Demographic and Health Survey. In this paper, we review the HIV Knowledge, Attitudes and Behaviour component of the 2011 Ugandan Demographic and Health Survey (UDHS) [26]. From the 2011 UDHS, a series of questions relating to HIV/AIDS was analysed comparing persons with and without disabilities in relation to HIV/AIDS knowledge, attitudes and practices. We believe that this analysis is among the first to use HIV/AIDS-related questions from Demographic Health Surveys (DHS) to provide information about persons with disabilities in comparison to those without disability and the general population.

## Method

### Demographic and health surveys

The worldwide Demographic and Health Surveys Program has collected, analyzed, and disseminated accurate and representative data on population, health, HIV, and nutrition through over 300 surveys in over 90 countries, and is primarily funded by the U.S. Agency for International Development (USAID) [27]. The Demographic and Health Surveys Program also supports AIDS Indicator Surveys (AIS), which provide countries with a standardized tool to obtain indicators for effective monitoring of national HIV/AIDS programmes [28]. In Uganda, DHS are important sources of information on HIV/AIDS and provide strategic information used to direct programmes and resources [29].

### Data

This study utilises secondary data from the 2011 UDHS [26], the fifth and most recent in a series of nationally representative surveys, which collected information on demographic, health, and family planning status and trends in Uganda. Permission to use the data was acquired through the DHS Program website [27]. The 2011 UDHS collected information on marriage; sexual activity; fertility preferences; awareness and use of family planning methods; infant, child, adult, and maternal mortality; awareness and behaviour relating to HIV/AIDS and other STDs; and levels of anaemia and vitamin A deficiency. Information was also collected on the extent of disability using the Washington Group Short Set, whereby participants reported difficulties that they may have doing certain activities due to a health problem in relation to seeing; hearing; walking or climbing steps; remembering or concentrating; self-care; and communicating, on a continuum of no difficulty; some difficulty; a lot of difficulty; and cannot do at all [30].

The 2011 UDHS is a nationally representative survey of 10,086 households with 9,247 women aged 15–49 and 2,573 men aged 15–54. Notably, the 2011 UDHS reports a 19.2% rate of disability for those aged 5 years and older [26], an estimate above that reported in the World Report on Disability of approximately 15% of the world’s population [31]. The sample was selected in two stages: the first stage was comprised of 404 enumeration areas selected from a list of clusters from the 2009/10 Uganda National Household Survey (UNHS) [32]. Matching of samples was conducted in this way to enable linking of the 2011 UDHS health indicators to poverty data from the 2010 UNHS. In the second sampling stage, households from each cluster were purposively selected from an updated complete listing of households. The UDHS sample included all households from the 2010 UNHS that were in the 404 enumeration areas.

The 2011 UDHS was comprised of four questionnaires: Household Questionnaire; Woman’s Questionnaire; Maternal Mortality Questionnaire; and Man’s Questionnaire. The Household Questionnaire was used to list all usual household members and visitors who spent the night before in the selected households. All women aged 15–49 who were either permanent residents of the households or visitors who slept in the households on the night prior to the survey were eligible to be interviewed using the Woman’s Questionnaire. Furthermore, in a subsample of one-third of households selected for the survey, all men aged 15–54 were eligible to be interviewed for the Man’s Questionnaire if they were either permanent residents or visitors who slept in the household on the night prior to the survey. The Man’s Questionnaire collected similar information to the Woman’s Questionnaire but was shorter as it did not include a detailed reproductive history or questions relating to maternal and child health. The Maternal Mortality Questionnaire was administered to all eligible women aged 15–49 in 35 additional households in 394 of the 404 enumeration areas. Detailed information in relation to the survey objectives, design, survey instruments, pre-testing, training and fieldwork are available in the survey main report [26].

The current study utilises data from all four questionnaires used in the 2011 UDHS. The 10,086 sampled households had a total of 9,480 occupied households. Of these, 9,033 households were interviewed (95% response rate), comprising 2,295 males aged 15–54 years old and 8,674 women aged 15–49 years old. The Household Questionnaire covered respondents’ demographic characteristics (e.g. age, sex, education, wealth, and disability), general household characteristics, and household population. The household member data was used for descriptive characteristics of household members by disability status.

The Woman’s and Man’s Questionnaires explored a wide range of topics including marriage and sexual activity; adult mortality; gender-based violence; and awareness and behaviour with regards to AIDS and other STDs. To explore HIV knowledge and disability, the household members data was merged with individual level data for males (N = 2,295) and individual level data for females (N = 8,674). The complete merged data for males and females resulted in a sample size of 10,969 individuals used in this study analyses. Data was merged following instructions from the *Guide to DHS Statistics* [33].

### Data analyses

The report entitled *Description of the Demographic and Health Surveys Individual Recode Data File* by MEASURE DHS was studied carefully by the authors in order to generate appropriate and relevant variables for purposes of the analysis presented in this study [34]. Analysis was conducted in two steps using STATA 12.1 [35]. The first step explored the impact of the explanatory variables on the outcome variables using bivariate analysis. The second step involved multivariate analysis; ordinary least squares regressions for continuous outcome variables; and logistic regression for binary outcome variables. The regressions included only explanatory variables that had a significant impact of 10% level of significance on the outcome variable for the bivariate regressions. All analyses included clustering at the household level to avoid downward bias in standard errors and overstating of *t* statistics [36]. The main outcomes of interest were HIV/AIDS knowledge, transmission and prevention methods, and sexual behaviour.

The main explanatory variable was disability, generated from the Washington Group Short Set [24,26,30]. Questions selected from the 2011 UDHS for this study related to the outcomes of interest, as outlined in S1 Table. Disability was defined as (i) any disability type for individuals with at least some difficulty in any of the functional areas; (ii) single disability for individuals with disability in only one functional area; (iii) multiple disability for individuals with disability in two or more functional areas; (iv) low severity of disability for individuals with only ‘some difficulty’ in one of the functional areas; (v) high severity of disability for individuals with ‘a lot of difficulty’ or ‘cannot do at all’ in one of the functional areas; and (vi) hearing disability for individuals with difficulty in hearing even when using a hearing aid.

We looked at the Deaf population as a distinct group to see if we might identify distinct disability-specific patterns due to the asserted difference between Deaf and hearing populations in relation to HIV in much of the literature [37–40]. In relation to Uganda, it is proposed that because the Deaf population lack access to healthcare and experience barriers to effective health literacy, this population may be at increased risk for HIV/AIDS [41].

All regression models controlled for the following explanatory variables: age (continuous years); education (none; primary; secondary; and higher); marital status (never married; married; and separated/divorced or widowed); wealth index (poorest; poorer; middle; richer; and richest); residence type (urban; semi-urban; and rural); and gender (male = 1; and female = 0). The DHS wealth index is a composite measure of households’ cumulative living standards, and classifies households into five wealth quintiles. The wealth index is measured using data on households’ ownership of selected assets, such as bicycles; materials used for housing construction; and types of water access and sanitation facilities [42].

## Results

Table 1 provides an overview of respondents’ descriptive characteristics by disability status. On average, persons with disabilities were generally older (32 years compared to 27 years); had no formal education (16% compared to 13%); had been previously married (17% compared to 9%); reported being poorer (21% compared to 16%); and resided in a rural area (77% compared to 70%).

**Table 1:** Descriptive characteristics by disability status.

Variable	All [mean, (SD)]	Persons with a disability	Persons without a disability	P Value
CONTROL VARIABLES				
**Age (years):**	28.14 (9.64)	32.54 (10.68)	27.26 (9.17)	0.000**
**Education:**				
No Education (N = 10,969)	13.24 (1,451)	16.25 (296)	12.65 (1,155)	0.000**
Primary	56.21 (6,158)	60.70 (1,106)	55.31 (5,052)	0.000**
Secondary	23.41 (2,565)	18.33 (334)	24.43 (2,231)	0.000**
Higher	7.14 (782)	4.72 (86)	7.62 (696)	0.000**
Education years	6.01 (4.16)	5.24 (3.96)	6.16 (4.18)	0.000**
**Marital Status:**				
Never married	28.93 (3,170)	19.65 (358)	30.78 (2,812)	0.000**
Currently married	60.58 (6,638)	63.23 (1,152)	60.05 (5,486)	0.011*
Formerly married	10.49 (1,149)	17.12 (312)	9.16 (837)	0.000**
**Wealth Index:**				
Poorest	19.67 (2,156)	19.86 (362)	19.63 (1,794)	0.825
Poorer	16.79 (1,840)	20.63 (376)	16.02 (1,464)	0.000**
Middle	16.27 (1,783)	19.03 (347)	15.71 (1,436)	0.000**
Richer	18.46 (2,023)	18.71 (341)	18.41 (1,682)	0.764
Richest	28.82 (3,159)	21.78 (397)	30.23 (2,762)	0.000**
**Residence Type:**				
Urban	23.13 (2,535)	17.55 (320)	24.24 (2,215)	0.000**
Semi-urban	5.98 (656)	5.76 (105)	6.03 (551)	0.657
Rural	70.89 (7,770)	76.69 (1,398)	69.73 (6,372)	0.000**
Male* (Note that males interviewed were 2,295/10,969)	20.91 (2,292)	21.45 (391)	20.80 (1,901)	0.536

* Significant at the 0.05 probability level.

** Significant at the 0.001 probability level.

Table 2 outlines HIV/AIDS awareness, knowledge and prevention by disability status for respondents. Regression Model 1 includes variables that relate to HIV/AIDS awareness, knowledge of prevention including prevention of mother to child transmission, and rejection of misconceptions about HIV/AIDS. Regression Model 2 considers HIV/AIDS knowledge and sexual behaviour.

**Table 2:** HIV/AIDS awareness, knowledge and prevention by disability status.

Variable	All (%, n)	Persons with a disability	Persons without a disability	P Value
OUTCOME VARIABLES				
**Model 1: Awareness; Knowledge of Prevention; and Rejection of Misconceptions about HIV/AIDS:**				
**Model 1a:**				
RR = Reduced risk of HIV infection:				
RR using condom	85.34 (8,542)	87.21 (1,479)	84.96 (7,063)	0.017**
RR having one sexual partner	92.60 (9,803)	93.62 (1,657)	92.40 (8,146)	0.075*
Healthy person can be HIV infected	89.80 (9,487)	91.29 (1,624)	89.50 (7,863)	0.023**
R = Risk of HIV infection:				
R mosquito bites	30.60 (2,924)	34.39 (540)	29.86 (2,384)	0.000****
R share food	15.53 (1,580)	17.03 (282)	15.23 (1,298)	0.065*
OK for a person with HIV to teach	74.95 (7,910)	71.23 (1,258)	75.69 (6,652)	0.000****
OK to care for a relative with HIV in household	89.42 (9,633)	91.53 (1,643)	89.00 (7,990)	0.001***
OK to buy vegetables from a vendor with HIV	73.13 (7,930)	71.75 (1,293)	73.41 (6,637)	0.147
**Model 1b: Knowledge of Prevention of Mother-to-Child Transmission:**				
HT = HIV transmission possible during:				
HT pregnancy	71.17 (7,261)	74.24 (1,271)	70.55 (5,990)	0.002***
HT delivery	93.37 (9,654)	93.89 (1,628)	93.26 (8,026)	0.339
HT breastfeeding	90.51 (9,169)	89.91 (1,497)	90.63 (7,672)	0.358
**Model 2: HIV Knowledge and Sexual Behaviour:**				
Months since last HIV test	24.722 (35.91)	25.35 (36.44)	24.59 (35.80)	0.492
Received test results most recent HIV test	94.54 (7,347)	93.54 (1,231)	94.75 (6.116)	0.079*
Age at first sex	16.69 (3.06)	16.43 (3.00)	16.74 (3.07)	0.0003****
Condom last sex	14.01 (1,099)	13.69 (183)	14.07 (916)	0.710
Had genital sore in last 12 months	11.35 (1,241)	15.71 (286)	10.47 (955)	0.000****
Had genital discharge in last 12 months	9.98 (1,092)	13.41 (244)	9.30 (848)	0.000****
Had STD in last 12 months	11.16 (1,133)	14.48 (1,671)	10.51 (891)	0.000****
Can get condom	69.73 (5,862)	70.04 (961)	69.67 (4,901)	0.781
Number of sexual partners in last 12 months	1.31 (4.69)	1.40 (5.35)	1.30 (4.54)	0.521
Lifetime sexual partners	3.00 (6.17)	3.61 (7.56)	2.87 (5.83)	0.0003****

* Significant at the 0.1 probability level.

** Significant at the 0.05 probability level.

*** Significant at the 0.01 probability level.

**** Significant at the 0.001 probability level.

**Fig 1:**
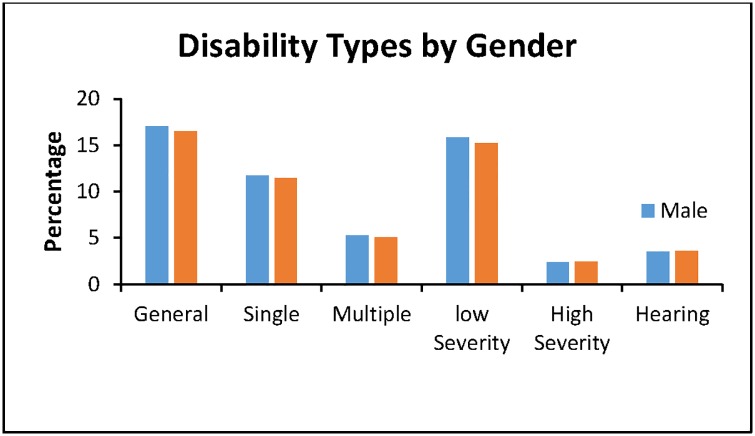
Disability types by age groups.

The proportion of the sample by different disability categories is shown in Table 3. 16.6% had any type of disability, i.e. at least some difficulty in one of the domains; 11.5% had a single disability; and 5.1% had multiple disabilities. 15.4% had low severity and 2.5% had high severity. 3.6% reported a hearing disability. As outlined in Table 3, there were no significant differences between genders for any of the disability types. As expected, disability was more prevalent in older individuals (Fig 1).

**Table 3:** Disability categories by gender for 15–54 year olds.

Explanatory Variable	All % (n)	Males	Females	*P* Value
Any disability type	16.6 (1,823)	17.1 (391)	16.5 (1,432)	0.536
Single disability	11.5 (1,259)	11.8 (269)	11.4 (990)	0.674
Multiple disability	5.1 (558)	5.3 (121)	5.1 (437)	0.646
Low severity	15.4 (1,682)	15.8 (363)	15.2 (1,319)	0.462
High severity	2.5 (268)	2.4 (55)	2.5 (213)	0.874
Hearing disability	3.6 (390)	3.5 (80)	3.6 (310)	0.874

### Multivariate analysis

Multivariate Logistic Regression Models are outlined in S2–S7 Tables. A summary of the multivariate analysis is shown in Table 4 (note that empty cells represent non-significant results). Notably, persons with disabilities had comparable levels of HIV/AIDS information with a few exceptions. However, they were more likely to indicate risk of HIV infection through mosquito bites and sharing food; less likely to report that it is okay to buy vegetables from a HIV-infected vendor (multiple disability); and less likely to approve of a healthy person with HIV/AIDS being a teacher (Table 4).

**Table 4:** Multivariate analysis: HIV/AIDS awareness, knowledge and sexual behaviour results.

Main Explanatory Variable	Any Disability Type AOR/Coeff [CI]	Single Disability AOR/Coeff [CI]	Multiple Disability AOR/Coeff [CI]	Low Severity AOR/Coeff [CI]	High Severity AOR/Coeff [CI]	Hearing Disability AOR/Coeff [CI]
OUTCOME VARIABLES						
**Model 1: Awareness; Knowledge of Prevention; and Rejection of Misconceptions about HIV/AIDS:**						
**Model 1a:**						
Heard of AIDS					0.20^b^ [0.06–0.74)	
RR using condom	1.28^a^ [1.09–1.50]	1.22^b^ [1.01–1.46]	1.32^b^ [1.00–1.72]	1.28^a^ [1.09–1.51]		
RR having one faithful sexual partner	1.20^c^ [0.97–1.48]	1.26^c^ [0.98–1.61]				
Healthy person can be HIV infected	1.21^b^ [1.00–1.46]	1.29^b^ [1.03–1.61]		1.21^c^ [0.99–1.47]		
R mosquito bites	1.15^b^ [1.02–1.30]		1.31^b^ [1.08–1.60]	1.16^b^ [1.02–1.31]		
R share food	1.15^c^ [0.99–1.34]				1.39^b^ [1.00–1.93]	
OK for a person with HIV to teach	0.84^a^ [0.74–0.95]		0.70^a^ [0.58–0.86]	0.83^a^ [0.73–0.94]		
OK to care for a relative with HIV	1.27^b^ [1.05–1.53]	1.22^c^ [0.97–1.53]		1.24^b^ [1.02–1.51]		
OK to buy vegetables from a vendor with HIV			0.74^a^ [0.61–0.91]			0.82^c^ [0.65–1.03]
**Model 1b: Knowledge of HIV/AIDS Mother-to-Child Transmission:**						
HIV transmission possible during pregnancy	1.22^a^ [1.08–1.38]	1.22^b^ [1.06–1.41]		1.19^b^ [1.05–1.35]		
**Model 2: HIV/AIDS Knowledge and Sexual Behaviour:**						
Months since last HIV test (OLS)	-0.68^b^ [-1.19–0.16]		-0.90^b^ [-1.81–0.01]	-0.70^b^ [-1.23- -0.2]		-1.31^b^ [-2.34- -0.28]
Received test results for most recent HIV test	0.79^c^ [0.61–1.02]		0.60^b^ [0.41–0.87]	0.76^b^ [0.59–0.99]	0.60^c^ [0.35–1.01]	
Age at first sex (OLS)	-0.34^a^ [-0.50- -0.18]	-0.17^b^ [-0.36–0.01]	-0.61^a^ [-0.88- -0.34]	-0.37^a^ [-0.53- -0.2]	-0.43^b^ [-0.86- -0.004]	
Used condom last sex	1.32^b^ [1.08–1.61]		1.60^b^ [1.15–2.22]	1.33^b^ [1.09–1.63]		1.81^a^ [1.24–2.62]
Had genital sore in last 12 months	1.48^b^ [1.27–1.72]	1.31^a^ [1.10–1.56]	1.62^a^ [1.28–2.07]	1.49^a^ [1.27–1.75]	1.34^c^ [0.95–1.89)	1.63^a^ [1.22–2.18]
Had genital discharge in last 12 months	1.43^a^ [1.21–1.68]	1.20^c^ [0.99–1.45]	1.73^a^ [1.34–2.23]	1.45^a^ [1.23–1.72]	1.55^b^ [1.07–2.23]	1.48^b^ [1.09–2.00]
Had STD in last 12 months	1.38^a^ [1.18–1.63]	1.26^b^ [1.05–1.51]	1.48^a^ [1.14–1.93]	1.32^a^ [1.11–1.56]	1.67^b^ [1.16–2.41]	1.51^b^ [1.12–2.04]
Can get condom						
Number of sexual partners in last 12 months (OLS)					-0.26^a^ [-0.4- -0.08]	-0.27^a^ [-0.43- -0.12]
Total lifetime sexual partners (OLS)	0.34^c^ [-0.01–0.68]	0.40^c^ [-0.04–0.84]		0.39^b^ [0.02–0.75]		

a, b and c represent p<0.01, p<0.05 and p<0.10 respectively; AOR = Adjusted Odds Ratio; CI = 95% confidence interval; Coeff = coefficient; OLS = ordinary least squares regression; R = Risk of HIV infection; RR = Reduced risk of HIV infection; STD = Sexually transmitted disease. Empty cells indicate non-significant findings.

Generally, persons with disabilities had fewer days since last HIV test compared to persons without a disability. For example, persons with any type of disability had 20 days fewer since the last HIV test compared to individuals without a disability. Persons with multiple disability had 27 days fewer; persons with low severity had 21 days fewer; while persons with a hearing disability had 39 days fewer since the last HIV test compared to individuals without a disability. However, persons with disabilities were statistically significantly less likely to return to the test clinic for HIV/AIDS test results compared to persons without a disability.

Also of particular note, persons with disabilities reported having a first sexual encounter at a younger age. However, the difference was minor, with a first sexual encounter generally earlier by only a few months, not years. Persons with any type of disability reported a first sexual encounter at 4 months younger than persons without a disability (*p* < 0.01). Individuals with a single disability reported a first sexual encounter at 2 months younger (*p* < 0.05); 7 months earlier for persons with multiple disabilities (*p* < 0.01); 4.5 months younger for individuals with low severity disability (*p* < 0.01) and 5 months younger for persons with high severity disability (*p* < 0.05). This difference was therefore greatest for persons with multiple disabilities.

Of particular concern, persons with disabilities were more likely to have reported having a STD in the last 12 months, statistically significantly higher than for persons without a disability (Table 4, Model 2). For example, individuals with multiple disabilities and persons with a hearing disability were almost twice as likely to have reported genital sores in the last 12 months compared to persons without a disability. Individuals with multiple disabilities and high severity disability were almost twice as likely to have reported a genital discharge in the last 12 months; while persons with high severity disability and a hearing disability were almost twice as likely to have reported a STD in the last 12 months compared to persons without a disability. This statistical significance was consistent for persons with all levels of disability assessed (any disability type, single, multiple, low and high severity) and disability types assessed (hearing disability); with odds greater than 1 for all disability categories compared to persons without a disability.

## Discussion

Our results present an evolving pattern of understanding and actions surrounding persons with disabilities and HIV and AIDS in Uganda. Comparing persons with disabilities with members of the population without a disability, we found a solid knowledge base of HIV/AIDS among a significant part of the population of persons with disabilities, which may reflect both greater AIDS outreach efforts to the general population and specific efforts to provide more information on HIV/AIDS to persons with disabilities in recent years. While we have no large baseline against which we can compare changes over time, these new figures may indicate that current outreach efforts are making progress.

Accordingly, our findings are contradictory to those of earlier and more recent studies, which found that persons with disabilities are less knowledgeable than those without disabilities in Uganda [2,6] and elsewhere [9,10,31,43–45]. Incongruity between our findings and those of previous studies could be a result of diverse methodologies returning different results. This is among the first times that a DHS has identified persons with disabilities using the Washington Group Short Set. These disability measures identify persons with disabilities in a markedly different way relative to how other studies identify persons with disabilities. As outlined in the World Report on Disability [31], operational measures of disability contrast in relation to the aim and application of the data; how disability is conceptualized; the features of disability that are assessed including impairments, restrictions on participation and environmental factors; definitions; design of questions; reporting sources; methods of data collection; and expectations of functioning.

It is also of note that while persons with and those without disabilities reported largely the same level of knowledge, several gaps in the knowledge base of persons with disabilities appear to continue, for example, misconceptions about the risk of HIV infection through mosquito bites and sharing food. Interestingly, although some literature indicates that the Deaf population may have higher rates of misinformation because of issues related to translating HIV/AIDS messages into local sign languages [39,44,45], in our study when the Deaf community was separated during analyses, Deaf individuals did not have higher rates of misconceptions around transmissions facts than did the broader disability community.

One incongruous finding from this study was that as a group, persons with disabilities reported greater knowledge than those without disabilities about the need to use condoms to reduce risk of HIV (AOR 1.32, *p* < 0.05). However, our findings also indicated a higher incidence of reported STDs for persons with disabilities. If persons with disabilities reported a higher rate of knowledge about condom usage and comparable rate of actual use, it is unclear why this group would have a higher reported rate of STDs. STDs are a marker of unprotected sexual intercourse and a co-factor in HIV transmission [26]. It is possible that some persons with disabilities may have long-term STDs and have elected, or are encouraged by partners, to use condoms, which would produce a higher knowledge level even if practices are not consistent and hence our findings indicate a higher incidence of reported STDs for persons with disabilities. It is also possible that while persons with disabilities report a higher rate of condom knowledge relating to HIV and comparable rate of condom usage relative to persons without disabilities, they are not receiving adequate information about effective condom use. It is also possible that lack of accessible places to buy condoms, embarrassment or harassment while buying condoms or poverty associated with disabilities makes it harder for persons with disabilities to purchase condoms [46]. Furthermore, most significantly, these higher rates may point to lack of ability to negotiate safer sex practices with partners among persons with disabilities no matter what their level of knowledge [14].

Although the discrepancy was not great–months rather than years–we also found that persons with disabilities reported becoming sexually active earlier than their peers without disabilities. Interestingly, these findings were true for both males and females. There may be a number of reasons for this finding, including targeted abuse by those without disabilities; persons with disabilities being anxious to prove that they are just like everyone else; or persons with disabilities being anxious to prove to the data collectors that they are just like everyone else and overstating their early sexual activity. Although often treated as asexual, a number of researchers and advocates have called attention to the fact that persons with disabilities may be more susceptible to being targeted for, or suffer from low self-esteem that make them at risk for, abuse and exploitation at an early age [13,14,47–50]. In a qualitative study by Yousafzai *et al*. [6], Ugandan and Rwandan adolescents with disabilities reported low self-esteem and self-efficacy, which impacted on control over safe sexual relationships; a substantial level of exploitation, targeted abuse, and rape were reported. In this study, while age at first sexual encounter was slighted earlier but comparable to peers without a disability, we did not ask the nature of this first encounter and this may also differ. In a study of knowledge, attitudes, and practices of persons with disabilities in Uganda relating to reproductive health and HIV/AIDS, 22% of women with disabilities reported that they were raped at their first sexual encounter [51]. Our findings, in conjunction with the findings from Mulindwa, indicate that further study on this issue is warranted.

A key finding is that persons with disabilities were as likely to be tested for HIV as the rest of the population but were significantly less likely than persons without disabilities to return to receive their test results. Persons with disabilities often, although not always, have health needs originating from their primary condition, and therefore on average may have more health needs than the majority of the population [15,52,53]. Accordingly, more inpatient and outpatient care is sought by persons with disabilities than those without disabilities [31,54]. While disability is not a ‘health problem’, some persons with disabilities do have increased health needs [55,56]. Persons with disabilities are likely to be seeking healthcare. Hence, it is unclear why our findings indicate that persons with disabilities are less likely to return to receive their HIV test results. We hypothesise several reasons for this, outlined below.

Transportation to clinics may be acting as a barrier for persons with disabilities regarding their ability to return to receive test results. A growing body of literature indicates that lack of accessible transportation and expense of transportation among a group who are routinely disproportionately poor may limit the ability of persons with disabilities to return for test results compared to their peers without disabilities [57]. Nixon *et al*. [19] reported the challenges experienced by persons with mobility or visual impairments in Zambia, who frequently required assistance to travel to clinics for testing, which resulted in double transportation costs for an assistant, or the need to request favours from neighbours.

Physical access to clinics is often discussed as a barrier to receiving test results for persons with disabilities [12,58,59]. In a qualitative study conducted by Tun and colleagues [60], one of the most significant barriers to accessing facility-based HIV services for persons with disabilities in Uganda was related to physical accessibility to and of HIV services facilities. Presumably persons with disabilities who come for an initial test have already found the local clinical services accessible, but if results are provided someplace other than where the initial testing took place, accessibility may be an issue.

Inaccessible formats of HIV test results may be presenting as a barrier to the receiving of HIV test results by persons with disabilities. In a qualitative study of perceptions of HIV-related health services for persons with disabilities who are HIV-positive in Zambia [19], numerous participants reported stories of individuals being denied testing due to healthcare workers experiencing communication constraints. Yousafzai *et al*. [45], in a qualitative study of perceptions of HIV/AIDS amongst persons with disabilities in Swaziland, reported apprehension by persons with disabilities relating to clinical visits due to communication, social, and physical barriers. Accordingly, simplified formats, pictures, Braille, or sign interpreters may be required [12,59].

Healthcare workers may place lower priority for good quality care on ensuring follow-up care for persons with disabilities when there are limited HIV services and medications available [12]. If persons with disabilities are not treated with dignity and their privacy ensured when they come for their initial tests, they may be reluctant or unwilling to return for the results. For example, in a qualitative study by Yoshida and colleagues [50] on the experiences of persons with disabilities who had contracted HIV in Zambia, participants reported discrimination in relation to a lack of expectation of persons with disabilities in HIV clinics. Yousafzai *et al*. [6], in a study of adolescents with disabilities in Rwanda and Uganda, reported that persons with disabilities were marginalized from HIV services as a result of health workers’ assumptions that they were not sexually active. It is also asserted that families and caregivers of persons with disabilities frequently do not adequately understand their sexual and reproductive health requirements [61], and this may result in, among other things, lack of encouragement by family members of persons with disabilities to return for follow-up results.

### Implications for practice

The key finding above is that while both persons with and without disabilities are presenting to be tested, there is a significant number of persons with disabilities who are failing to return to find out the results of their tests. Testing means little if people do not return for follow-up to know their HIV status and, if necessary, to be connected to services and supports that are available.

A further complicating factor may be that healthcare workers may not have the required information, skills, and resources to provide suitable and accessible HIV services for persons with disabilities, and more effective training and peer support may be essential [12,59,61,62]. Best practice interventions that include disability in HIV care need to be developed, assessed and made available to healthcare workers in the African context [63]. For example, HEARD’s healthcare workers project has designed a sensitization workshop, including knowledge sharing on the association between disability and HIV, which has since been adopted by the KwaZulu-Natal Department of Health [62]; and Hanass-Hancock and Mdletshe [64] have developed a facilitators’ guide for training for, and with, persons with disabilities on the association between disability and HIV.

To ensure access to HIV health services for individuals with disabilities in Uganda, two key principles of the UNCRPD can guide the design of interventions, structures and services: *universal design*, suggesting that the design of products, environments, programmes, and services is usable by all people to the greatest extent possible, such as providing a ramp to a health facility; and *reasonable accommodation*, signifying that necessary and appropriate modifications are made where needed for persons with disabilities to enjoy their rights on an equal basis with others, such as providing a wheelchair [7,12]. Including persons with disabilities in general AIDS campaigns and through disability-specific interventions is essential [65]. Inclusive and targeted HIV, sexual and reproductive health services are required, with the involvement of persons with disabilities [66].

### Implications for future research

While our findings indicate comparable levels of knowledge on HIV/AIDS for those with and without disabilities, which may reflect increased attention to reaching the community of persons with disabilities, it is important that future research determine if information is provided in disability-inclusive ways and that provisions are made for universal design, for example, Braille, audio formats, and sign language.

While knowledge, attitudes and behaviours from the 2011 UDHS have provided interesting insights into these variables broadly among persons with disabilities in Uganda, unfortunately, the 2011 Ugandan AIS does not presently collect data on persons with disabilities. This is a lost opportunity as a central aim of Uganda’s AIS is to obtain estimates of HIV prevalence, its risk factors, programme coverage, and indicators of knowledge, behaviours, and attitudes [29]. The inclusion of the Washington Group Short Set in Uganda’s AIS could provide cyclical, systematic and comprehensive data on disability and HIV/AIDS, including HIV prevalence amongst persons with disabilities. As proposed by Martin Babu [67], mainstreaming disability in the Ugandan HIV/AIDS National Response could enable disability inclusion to be advanced at the national level in all development processes. Such population-based surveys need to be conducted in a way that maximises the comparability, usefulness, and quality of data on HIV within countries [68].

If persons with disabilities are less likely to receive HIV test results, it is important that further research determine why. We hypothesise that not one but a series of factors, including formats for reporting test results; expenses and accessibility issues for a repeat trip; and limited knowledge of disability and HIV among healthcare workers, families, and caregivers, may all act as barriers to providing test results for persons with disabilities. Qualitative research would be valuable in exploring this issue in greater depth, particularly taking into account the social meaning of the disease, the social context in which reporting of test results is performed, and attributes of the healthcare system [69]. A linked collection of qualitative data through interview and focus groups might add greater depth to understanding why these discrepancies exist.

We found that persons with disabilities reported becoming sexually active slightly earlier than their peers without disabilities, although the discrepancy was not substantial–months rather than years. The nature of these first sexual encounters however may differ, with potentially higher rates of violence and coercion for persons with disabilities–and this warrants further investigation. In Uganda, qualitative studies, such as those conducted by Yousafzai *et al*. [6] and Mulindwa [51], could help to explore this issue in greater depth. Knowing that persons with disabilities surveyed reported a first sexual encounter at approximately the same age as those without disabilities, indeed a few months on average earlier, further underscores the importance of inclusion of persons with disabilities, both males and females, in all sexual and reproductive health campaigns aimed at the general public.

Higher reported rates of STDs within the past 12 months is of particular concern and again warrants in-depth exploration to identify patterns of risk, access to care and need for more effective outreach and intervention.

### Limitations

The 2011 UDHS does not explicitly outline how information was collected across persons with various types and severity of disability, including persons with learning, intellectual, mental health, speech and language, physical, visual or hearing impairments. Interviewer protocols for implementing the Washington Group Short Set with persons with disabilities [70] recommend establishing the most effective communication method for the individual respondent and setting up the required supports to use that method prior to the interview, including the use of large print, Braille, sign-language, or interviewing the individual in the presence of an interpreter, facilitator, advocate or parent/guardian.

The Washington Group Short Set, used to identify persons with disabilities in this study, has inherent limitations. For example, clauses such as ‘even when wearing glasses’ when assessing seeing difficulty may create confusion amongst respondents; the questions also do not address psychological issues [71]. For such reasons, an extended set of questions is currently under development by the Washington Group [23,24,71]. While the Washington Group Short Set has been recommended for use in censuses and surveys, as part of the upcoming Sustainable Development Goals [72], it is of note that the Short Set may not be sensitive enough to capture specific health information for persons with different impairment types or levels of disabilities, particularly for those with mental health issues.

Indeed, the Washington Group Short Set is based on a narrow definition of disability, designed with the purpose of equalization of opportunities. As proposed by the Washington Group on Disability Statistics, the Short Set identifies persons with functional limitations that may limit independent participation in society. However, the Short Set does not identify all persons with limitations or is necessarily representative of the ‘true’ population with disability, which would necessitate assessing limitations in all domains using a more comprehensive set of questions [73].

Notably, the World Health Organization Disability Assessment Schedule 2.0 (WHODAS 2.0) provides a standardized method for measuring health and disability across cultures, and comprises six domains of functioning including cognition; mobility; self-care; getting along; life activities; and participation [74]. The WHODAS 2.0 provides disability questions short sets that overcome the limitations of the Washington Group Short Set, which does not take account of contextual factors; incorporating the six domains into the Washington Group Short Set enables measuring the lived experience of health beyond assessing the outcomes of diseases [75]. The WHODAS 2.0 has been used to measure disability for persons living with HIV. For example, Hanass-Hancock *et al*. [76] reported that 35.5% of 1,042 adult participants living with HIV in South Africa acquired a weighted score of two or more on the WHODAS 2.0, indicating potential activity limitations.

A further concern was that age of onset of disability, whether from birth or later in life, and cause of disability were not assessed and this may make a difference in knowledge, attitudes and practices in relation to HIV and AIDS [6,12,77]. Finally, the Washington Group Short Set uses self-reported disability measures. The primary disadvantage of self-reported measures is their subjectivity and dependence on individual understanding, perspectives and life contexts; and their reliance on respondents’ precision of understanding and choice of responses [78]. Despite the above limitations, this paper contributes to filling the knowledge gap, particularly because we have used nationally representative data, building beyond previous research that has used sample data [6,79].

## Conclusions

Uganda has a strong history of policy and programmes that support the rights of persons with disabilities. This is evident in Uganda’s national legal framework [80]. The Ugandan Constitution in Article 32 upholds that “the State shall take affirmative action in favour of groups marginalised on the basis of gender, age, disability or any other reason created by history, tradition or custom, for the purpose of redressing imbalances which exist against them” [81]. Disability is also recognized in the Uganda 2010 HIV Counselling and Testing Policy, which in Article 6.2 recognizes that persons with disabilities may be at higher risk of HIV infection and also may experience difficulty in accessing services. The Article stipulates that all HIV Counselling and Testing services address the unique needs of persons with disabilities [82].

In a review of eighteen national strategic plans for HIV and AIDS in Eastern and Southern Africa on the inclusion of persons with disabilities, Hanass-Hancock and colleagues [83] found that the Ugandan plan reflected best practice in the region as it recognized that persons with disabilities have been excluded in the response to HIV and AIDS and that HIV is increasingly a cause of disability. According to a World Bank study [2] of HIV/AIDS and disability in Uganda, Disabled People’s Organizations have also been very involved in the response to AIDS; for example, the National Union of Disabled Persons of Uganda [84], a domestic umbrella non-governmental organization of persons with disabilities, established the Disability Stakeholder HIV/AIDS Committee in 2005 [85], which has significantly contributed to coordinating the disability sector around HIV/AIDS.

Uganda is also one of the first countries to include the Washington Group Short Set in its national DHS. As proposed by Mitra [18], the adoption of the Washington Group Short Set in DHS activates the data revolution required to assess progress towards inclusive development and to monitor implementation of the UNCRPD–data crucial to assessing the inclusion, human rights and opportunities of populations with disabilities. However, the parallel inclusion of the Washington Group Short Set in Uganda’s AIS would provide valuable data relating to HIV and disability, including HIV prevalence for the population with disabilities. Such inclusion of disability measures in Uganda’s AIS would be in keeping with the requirements of the UNCRPD and its 2008 Optional Protocol [7], which Uganda has ratified [86], specifically its Article 31 on ‘Statistics and Data Collection’, specifying that “States parties undertake to collect appropriate information, including statistical and research data, to enable them to formulate and implement policies to give effect to the present Convention”.

Furthermore, while our findings suggest comparability between persons with disabilities and the remaining population in HIV knowledge and in testing rates, a key finding is that persons with disabilities were less likely to receive results from their most recent HIV test. Reports of slightly earlier age of first sexual experience and strikingly higher rates of having had an STD within the past 12 months among persons with disabilities compared to their peers without disabilities is of significant importance and further research is needed to provide insights into these differences and other identified discrepancies between those with and without disabilities in Uganda.

Our findings are consistent with the gap between laws, policies and practice regarding disability in Uganda discussed by Abimanyi-Ochom and Mannan [80]. According to the National Union of Disabled Persons of Uganda [85], only modest attention has been afforded to disability and HIV/AIDS in Uganda, and no national study to date has established the prevalence of HIV/AIDS among persons with disabilities. As contended by Murangira [1], data collected on persons with disabilities and HIV/AIDS in Uganda could be greatly strengthened. For example, cards provided to Voluntary Counselling and Testing clients indicate that statistics are not being collected on disability and HIV/AIDS, creating a gap in informed approaches relating to persons with disabilities and HIV/AIDS. There is a call for routine and comprehensive collection and analyses of systematic disability data in Uganda, with the aim of evaluating policies and performance, including with regards to equities in access to health [80]. As expressed by Murangira [1] (p.31):

We disabled people in Uganda are aware of the enormous commitment the country and various stakeholders have made in the struggle against HIV/AIDS and are aware of the enormous financial constraints faced in alleviating the pandemic. But, in the area of our national health policy and services delivery, we believe there has been a tremendous oversight of the needs of disabled people and the impact that HIV/AIDS has on them.

Calls both at a national and global level are evident for HIV policies and programmes that explicitly recognize the need for HIV and rehabilitation services to be inclusive of persons with diverse disabilities [50]. The response to HIV/AIDS will only be successful if disability is consistently included in all outreach efforts [14,48,61,87].

Findings from this Ugandan national DHS raise as many questions as answers–both about successes and gaps in reaching the population with disabilities, and in the comparability of methodologies being developed to help collect and analyse findings to measure HIV/AIDS knowledge, attitudes and practices for this large and long overlooked population.

## Supporting information

S1 Table Selected 2011 UDHS questions related to outcomes of interest. (PDF)

S2 Table Has disability results combined. (PDF)

S3 Table Hearing disability results combined. (PDF)

S4 Table High severity results combined. (PDF)

S5 Table Low severity results combined. (PDF)

S6 Table Multiple disability results combined. (PDF)

S7 Table Single disability results combined. (PDF)
