# Supplementary material for: HIV/AIDS knowledge, attitudes and behaviour of persons with and without disabilities from the Uganda Demographic and Health Survey 2011: Differential access to HIV/AIDS information and services
Source: PLoS One. 2017 Apr 13;12(4):e0174877. doi: 10.1371/journal.pone.0174877 (PMC5390986; doi:10.1371/journal.pone.0174877)
Supplement: S1 Table — (PDF) [file pone.0174877.s001.pdf]

QUESTIONS	CODING CATEGORIES
Have you ever heard of an illness called AIDS?	YES/NO
Can people reduce their chance of getting the AIDS virus by using a condom every time they have sex?	YES/NO/DON'T KNOW
Can people reduce their chance of getting the AIDS virus by having just one uninfected sex partner who has no other sex partners?	YES/NO/DON'T KNOW
Is it possible for a healthy-looking person to have the AIDS virus?	YES/NO/DON'T KNOW
Can people get the AIDS virus from mosquito bites?	YES/NO/DON'T KNOW
Can people get the AIDS virus by sharing food with a person who has AIDS?	YES/NO/DON'T KNOW
In your opinion, if a female teacher has the AIDS virus but is not sick, should she be allowed to continue teaching in the school?	SHOULD BE ALLOWED<br>SHOULD NOT BE ALLOWED<br>DON’T KNOW/NOT SURE/DEPENDS
If a member of your family became sick with AIDS, would you be willing to care for her or him in your own household?	YES/NO<br>DON’T KNOW/NOT SURE/DEPENDS
Would you buy fresh vegetables from a shopkeeper or vendor if you knew that this person had the AIDS virus?	YES/NO/DON'T KNOW
Can the virus that causes AIDS be transmitted from a mother to her baby:<br>During pregnancy?<br>During delivery?<br>By breastfeeding?	YES/NO/DON’T KNOW
How many months ago was your most recent HIV test?	MONTHS AGO
I don't want to know the results, but did you get the results of the test?	YES/NO
How old were you when you had sexual intercourse for the very first time?	NEVER HAD SEXUAL INTERCOURSE<br>AGE IN YEARS
The last time you had sexual intercourse, was a condom used?	YES/NO
During the last 12 months, have you had a genital sore or ulcer?	YES/NO/DON'T KNOW
During the last 12 months, have you had a bad-smelling abnormal genital discharge?	YES/NO/DON'T KNOW
During the last 12 months, have you had a disease which you got through sexual contact?	YES/NO/DON'T KNOW
If you wanted to, could you yourself get a condom?	YES/NO/<br>DON'T KNOW/UNSURE

In total, with how many different people have you had sexual intercourse in the last 12 months?	NUMBER OF PARTNERS LAST 12 MONTHS<br>DON’T KNOW
In total, with how many different people have you had sexual intercourse in your lifetime?	NUMBER OF PARTNERS IN LIFETIME<br>DON’T KNOW
