# Supplementary material for: HIV/AIDS knowledge, attitudes and behaviour of persons with and without disabilities from the Uganda Demographic and Health Survey 2011: Differential access to HIV/AIDS information and services
Source: PLoS One. 2017 Apr 13;12(4):e0174877. doi: 10.1371/journal.pone.0174877 (PMC5390986; doi:10.1371/journal.pone.0174877)
Supplement: S2 Table — (PDF) [file pone.0174877.s002.pdf]

Multivariate Logistic Model-HIV/AIDS Knowledge and awareness

	(2)<br>reduced risk of HIV infection using condom	(3)<br>reduced risk of HIV infection one partner	(4)<br>healthy looking person can have HIV	(5)<br>risk of HIV infection mosquito bites	(6)<br>risk of HIV infection share food	(7)<br>okay for a teacher with HIV to teach	(8)<br>okay to care for a relative with HIV	(9)<br>can buy vegetables from HIV infected vendor
Has disability	**1.280****	**1.199+**	**1.210***	**1.151***	**1.152+**	**0.835****	**1.269***	0.940
	(0.106)	(0.130)	(0.117)	(0.0722)	(0.0870)	(0.0520)	(0.123)	(0.0588)
Age (years)	**0.992+**	1.004	**1.008+**	**0.991****	**0.981*****	**1.009****	**1.030*****	**1.014*****
	(0.00391)	(0.00534)	(0.00481)	(0.00309)	(0.00391)	(0.00324)	(0.00506)	(0.00318)
Primary Education	**1.141+**	1.137	**1.486*****	**0.527*****	**0.499*****	**2.300*****	**2.757*****	**2.350*****
	(0.0767)	(0.104)	(0.126)	(0.0289)	(0.0349)	(0.137)	(0.261)	(0.133)
Secondary plus	**1.329***	1.193	**2.089*****	**0.229*****	**0.309*****	**6.182*****	**3.764*****	**5.431*****
	(0.164)	(0.210)	(0.395)	(0.0280)	(0.0446)	(0.980)	(0.887)	(0.779)
Currently married	**1.356*****	1.154	**1.612*****	**1.139+**	1.085	**1.215****	**1.293****	**1.120+**
	(0.112)	(0.127)	(0.149)	(0.0765)	(0.0883)	(0.0846)	(0.122)	(0.0754)
Formerly married	**1.485****	1.182	**2.025*****	1.137	1.149	**1.533*****	**1.599****	**1.275***
	(0.184)	(0.198)	(0.307)	(0.111)	(0.136)	(0.156)	(0.243)	(0.126)
Poorer	1.170	1.194	**1.586*****	0.932	0.874	1.306***	2.336***	1.404***
	(0.112)	(0.143)	(0.158)	(0.0697)	(0.0781)	(0.0975)	(0.223)	(0.101)
Middle	**1.506*****	**1.418****	**1.967*****	0.883	**0.773****	**1.497*****	**2.792*****	**1.505*****
	(0.151)	(0.177)	(0.211)	(0.0685)	(0.0728)	(0.117)	(0.288)	(0.113)
Richer	**1.736*****	**1.511****	**2.098*****	0.882	0.952	**1.540*****	**3.058*****	**1.566*****
	(0.175)	(0.193)	(0.219)	(0.0688)	(0.0866)	(0.122)	(0.325)	(0.117)
Richest	**1.660*****	**1.816*****	**3.330*****	**0.749****	**0.814+**	**1.937*****	**4.924*****	**1.819*****
	(0.184)	(0.274)	(0.482)	(0.0683)	(0.0877)	(0.184)	(0.712)	(0.164)
Semi-urban	1.017	**1.445+**	1.409	**1.472*****	1.050	**0.662****	1.095	**0.768***
	(0.147)	(0.314)	(0.306)	(0.168)	(0.146)	(0.0836)	(0.235)	(0.0888)
Rural	0.896	1.153	0.876	**1.248****	0.987	**0.721*****	0.887	**0.727*****
	(0.0842)	(0.142)	(0.112)	(0.0941)	(0.0879)	(0.0610)	(0.117)	(0.0589)
Male	0.998	1.122	**1.797*****	1.053	**0.846***	0.964	1.029	**1.477*****
	(0.0731)	(0.112)	(0.173)	(0.0613)	(0.0632)	(0.0576)	(0.0900)	(0.0908)
ll	-4115.739	-2766.251	-3270.132	-5606.769	-4256.123	-5496.876	-3220.506	-5826.260
chi2	100	53	341	445	222	629	584	700
df_m	13.000	13.000	13.000	13.000	13.000	13.000	13.000	13.000
aic	8259.477	5560.501	6568.264	11241.537	8540.246	11021.752	6469.012	11680.521
bic	8360.426	5662.231	6669.966	11341.836	8641.427	11123.440	6570.987	11782.587
Observations	10003	10577	10556	9549	10170	10545	10764	10834

Odds Ratios; Standard errors in parentheses; Note: no education, never married, poorest, urban residence and female are controls for education, marital status, wealth status, residence type and gender dummies; N=Number of observations; + p<.10, * p<.05, ** p<.01, *** p<.001

Multivariate Logistic Model-HIV/AIDS transmission and disability

	(1) HIV transmission possible during pregnancy	(2) HIV transmission possible during delivery	(3) HIV transmission possible during breastfeeding	(4) Months since last HIV test (OLS)	(5) Received last HIV test results
Has disability	**1.221****	1.063	0.933	**-0.676***	**0.787+**
	(0.0768)	(0.125)	(0.0885)	(0.263)	(0.102)
Age (years)	**0.988*****	**1.013***	0.999	**0.137*****	**1.023*****
	(0.00297)	(0.00647)	(0.00487)	(0.013)	(0.00716)
Primary Education	**0.747*****	**2.026*****	**1.248****	-0.347	**1.658*****
	(0.0395)	(0.207)	(0.102)	(0.228)	(0.198)
Secondary-plus Education	**0.633*****	**4.281*****	**1.678*****	**1.236*****	**3.071*****
	(0.0543)	(1.100)	(0.262)	(0.345)	(0.816)
Currently Married	1.065	**2.047*****	**1.536*****	**0.493+**	1.146
	(0.0679)	(0.233)	(0.151)	(0.275)	(0.168)
Formerly Married	1.120	**2.364*****	**1.519****	0.170	1.075
	(0.107)	(0.419)	(0.224)	(0.405)	(0.229)
Poorer	0.961	1.138	0.883	0.246	0.897
	(0.0766)	(0.135)	(0.101)	(0.333)	(0.133)
Middle	1.103	**1.573*****	0.848	0.172	1.056
	(0.0908)	(0.204)	(0.0975)	(0.334)	(0.171)
Richer	**1.141+**	**1.481****	0.902	0.025	1.250
	(0.0900)	(0.189)	(0.105)	(0.338)	(0.213)
Richest	**1.185+**	**1.970*****	1.141	**0.835***	1.035
	(0.108)	(0.314)	(0.162)	(0.374)	(0.191)
Semi-Urban residence	0.865	0.758	1.032	-0.358	0.833
	(0.0859)	(0.156)	(0.182)	(0.434)	(0.209)
Rural Residence	**1.217****	0.848	0.887	0.181	**0.660***
	(0.0888)	(0.119)	(0.103)	(0.297)	(0.110)
Male	**0.713*****	1.124	**0.502*****	**-0.666***	**0.692****
	(0.0394)	(0.115)	(0.0387)	(0.260)	(0.0919)
Constant				**5.922*****	
				**(0.480)**	
Observations	10194	10332	10122	7772	7766

Odds Ratios (except for OLS regressions, coefficients); Standard errors in parentheses; "Note: no education, never married, poorest, urban residence and female are controls for education, marital status, wealth status, residence type and gender dummies; N=Number of observations; Odds Ratios." + p<.10, * p<.05, ** p<.01, *** p<.001

Multivariate Regression Model-HIV/AIDS Knowledge and Sexual Behaviour and disability

	(1) Age first sex	(2) last sex used condom	(3) genital sores l12M	(4) genital discharge l12M	(5) STD l12M	(6) can get condom	(7) number of partners l12M	(8) total number of lifetime sexual partners
Has disability	**-0.341*** **	**1.321** **	**1.476*** **	**1.425*** **	**1.384*** **	1.063	0.020	**0.335+**
	(0.084)	(0.133)	(0.116)	(0.119)	(0.115)	(0.0751)	(0.161)	(0.178)
Age (years)	**0.039*** **	**0.983** **	0.995	0.996	**0.989** **	**0.984*** **	0.013	**0.070*** **
	(0.004)	(0.00550)	(0.00386)	(0.00416)	(0.00407)	(0.00346)	(0.009)	(0.009)
Primary Education	**0.943*** **	**1.722*** **	0.931	1.058	1.037	**1.323*** **	**-0.225+**	0.021
	(0.072)	(0.149)	(0.0688)	(0.0807)	(0.0775)	(0.0758)	(0.117)	(0.165)
Secondary plus	**3.168*** **	**1.701*** **	**0.760***	**0.734***	0.836	**2.833*** **	-0.072	-0.388
	(0.139)	(0.225)	(0.106)	(0.113)	(0.115)	(0.305)	(0.272)	(0.340)
Currently married	**0.561*** **	**0.100*** **	**3.317*** **	**3.006*** **	**4.689*** **	**2.593*** **	-0.049	**0.569** **
	(0.100)	(0.0102)	(0.357)	(0.344)	(0.554)	(0.186)	(0.159)	(0.176)
Formerly married	0.092	**0.589*** **	**3.174*** **	**3.188*** **	**4.293*** **	**2.850*** **	0.513	**1.293*** **
	(0.134)	(0.0789)	(0.441)	(0.464)	(0.658)	(0.291)	(0.364)	(0.281)
Poorer	**-0.326** **	**1.317+**	**1.736*** **	**1.715*** **	**1.488** **	**1.340** **	0.180	**0.388***
	(0.102)	(0.218)	(0.200)	(0.219)	(0.199)	(0.125)	(0.172)	(0.152)
Middle	**-0.360*** **	**1.728*** **	**2.390*** **	**2.781*** **	**2.554*** **	**1.409*** **	-0.041	**0.534** **
	(0.102)	(0.267)	(0.268)	(0.344)	(0.326)	(0.132)	(0.123)	(0.165)
Richer	**-0.588*** **	**1.924*** **	**2.368*** **	**2.447*** **	**2.554*** **	**1.411*** **	0.073	**0.906*** **
	(0.107)	(0.290)	(0.268)	(0.309)	(0.330)	(0.128)	(0.155)	(0.195)
Richest	**-0.350** **	**2.010*** **	**2.113*** **	**2.473*** **	**2.500*** **	1.156	0.246	**1.150*** **
	(0.120)	(0.333)	(0.271)	(0.342)	(0.354)	(0.117)	(0.154)	(0.252)
Semi-urban	**-0.347***	1.000	1.083	1.002	1.011	1.045	0.553	-0.184
	(0.140)	(0.145)	(0.160)	(0.148)	(0.142)	(0.124)	(0.435)	(0.269)
Rural	0.128	**0.763***	1.121	0.989	1.029	**0.709*** **	-0.077	**-0.537***
	(0.100)	(0.0861)	(0.107)	(0.0965)	(0.0998)	(0.0550)	(0.123)	(0.234)
Male	**0.949*** **	**1.707*** **	**0.473*** **	**0.361*** **	**0.520*** **	**6.493*** **	0.092	**4.742*** **
	(0.088)	(0.144)	(0.0465)	(0.0425)	(0.0505)	(0.514)	(0.084)	(0.274)
Constant	**14.593*** **						**0.892*** **	**-0.926***
	(0.160)						(0.254)	(0.388)
Adjusted R^2	0.128						0.002	0.119
ll	-2.14e+04	-2458.575	-3662.245	-3341.780	-3318.264	-4659.532	-2.33e+04	-2.91e+04
chi2		1148	308	320	338	785		
df_m	13.000	13.000	13.000	13.000	13.000	13.000	13.000	13.000
aic	42870.958	4945.150	7352.490	6711.561	6664.528	9347.063	46581.725	58286.502
bic	42969.921	5042.686	7454.678	6813.748	6765.670	9445.569	46679.292	58386.240
Observations	8680	7839	10929	10928	10142	8401	7856	9174

Odds Ratios; Standard errors in parentheses; Note: no education, never married, poorest, urban residence and female are controls for education, marital status, wealth status, residence type and gender dummies; N=Number of observations; Odds Ratios. + p<.10, * p<.05, ** p<.01, *** p<.001
