# Supplementary material for: HIV/AIDS knowledge, attitudes and behaviour of persons with and without disabilities from the Uganda Demographic and Health Survey 2011: Differential access to HIV/AIDS information and services
Source: PLoS One. 2017 Apr 13;12(4):e0174877. doi: 10.1371/journal.pone.0174877 (PMC5390986; doi:10.1371/journal.pone.0174877)
Supplement: S3 Table — (PDF) [file pone.0174877.s003.pdf]

Multivariate Logistic Model-Hearing Disability and HIV/AIDS Knowledge and awareness

	(2) reduced risk HIV infection using condom	(3) reduced risk HIV infection one partner	(4) healthy looking person can have HIV	(5) risk HIV infection mosquito bites	(6) risk HIV infection share food	(7) okay a teacher with HIV to teach	(8) okay care for a relative with HIV	(9) okay buy vegetables HIV infected vendor
Hearing disability	1.190	1.084	1.001	1.022	**1.258+**	0.920	1.123	**0.818+**
	(0.187)	(0.226)	(0.178)	(0.121)	(0.175)	(0.110)	(0.214)	(0.096)
Age (years)	0.994	1.006	**1.010***	**0.992****	**0.982*****	**1.007***	**1.031*****	**1.014*****
	(0.004)	(0.005)	(0.005)	(0.003)	(0.004)	(0.003)	(0.005)	(0.003)
Primary Education	**1.134+**	1.133	**1.480*****	**0.525*****	**0.498*****	**2.308*****	**2.745*****	**2.349*****
	(0.076)	(0.104)	(0.125)	(0.029)	(0.035)	(0.137)	(0.260)	(0.133)
Secondary plus	**1.313***	1.183	**2.065*****	**0.228*****	**0.308*****	**6.229*****	**3.718*****	**5.429*****
	(0.162)	(0.208)	(0.390)	(0.028)	(0.045)	(0.987)	(0.875)	(0.778)
Currently married	**1.340*****	1.144	**1.595*****	**1.129+**	1.080	**1.226****	**1.279****	**1.120+**
	(0.110)	(0.125)	(0.147)	(0.076)	(0.088)	(0.085)	(0.121)	(0.075)
Formerly married	**1.487****	1.187	**2.029*****	1.140	1.152	**1.528*****	**1.608****	**1.274***
	(0.184)	(0.199)	(0.307)	(0.111)	(0.136)	(0.156)	(0.244)	(0.126)
Poorer	**1.175+**	1.201	**1.597*****	0.937	0.876	**1.298*****	**2.356*****	**1.404*****
	(0.112)	(0.143)	(0.159)	(0.070)	(0.078)	(0.097)	(0.225)	(0.101)
Middle	**1.512*****	**1.423****	**1.978*****	0.888	**0.774****	**1.492*****	**2.811*****	**1.505*****
	(0.151)	(0.178)	(0.212)	(0.069)	(0.073)	(0.116)	(0.290)	(0.113)
Richer	**1.733*****	1.513**	2.099***	0.883	0.955	1.538***	3.061***	1.563***
	(0.175)	(0.194)	(0.219)	(0.069)	(0.087)	(0.122)	(0.326)	(0.117)
Richest	**1.650*****	**1.811*****	**3.321*****	**0.747****	**0.811+**	**1.945*****	**4.907*****	**1.821*****
	(0.183)	(0.273)	(0.481)	(0.068)	(0.088)	(0.185)	(0.709)	(0.164)
Semi-urban	1.022	**1.452+**	1.418	**1.475*****	1.052	**0.659*****	1.102	**0.768***
	(0.148)	(0.316)	(0.307)	(0.168)	(0.146)	(0.083)	(0.236)	(0.089)
Rural	0.897	1.154	0.877	**1.249****	0.986	**0.721*****	0.886	**0.728*****
	(0.0844)	(0.142)	(0.112)	(0.094)	(0.088)	(0.061)	(0.117)	(0.059)
Male	0.999	1.121	**1.797*****	1.054	**0.847***	0.965	1.031	**1.475*****
	(0.073)	(0.111)	(0.173)	(0.061)	(0.063)	(0.058)	(0.090)	(0.091)
Observations	10,002	10,576	10,555	9,548	10,169	10,544	10,763	10,833

Odds Ratios for logistic regressions and coefficients for OLS regressions; Standard errors in parentheses; Note: no education, never married, poorest, urban residence and female are controls for education, marital status, wealth status, residence type and gender dummies; lifetime sexual partners=total number of lifetime sexual partners; partners =total partners; l12mths=Last 12 months; N=Number of observations; + p<.10, * p<.05, ** p<.01, *** p<.001

Multivariate Logistic Model-Hearing Disability and HIV/AIDS transmission

	(1)<br>HIV transmission possible during pregnancy	(2)<br>HIV transmission possible during delivery	(3)<br>HIV transmission possible during breastfeeding	(4)<br>Months since last HIV test (OLS)	(5)<br>Received last HIV test results
Low severity	1.149	1.128	1.312	**-1.308***	0.829
	(0.144)	(0.258)	(0.271)	(0.527)	(0.205)
Age (years)	**0.990*****	**1.014***	0.998	**0.134*****	**1.021****
	(0.003)	(0.006)	(0.005)	(0.013)	(0.007)
Primary Education	**0.744*****	**2.026*****	**1.255****	-0.348	**1.663*****
	(0.039)	(0.207)	(0.103)	(0.228)	(0.199)
Secondary plus	**0.628*****	**4.277*****	**1.694*****	**-1.230*****	**3.099*****
	(0.054)	(1.099)	(0.264)	(0.345)	(0.823)
Currently married	1.056	**2.044*****	**1.548*****	**0.503+**	1.155
	(0.067)	(0.232)	(0.152)	(0.275)	(0.169)
Formerly married	1.122	**2.368*****	**1.518****	0.148	1.068
	(0.108)	(0.419)	(0.224)	(0.405)	(0.226)
Poorer	0.966	1.139	0.879	0.229	0.889
	(0.077)	(0.135)	(0.100)	(0.332)	(0.132)
Middle	1.104	**1.573*****	0.843	0.172	1.045
	(0.091)	(0.204)	(0.097)	(0.334)	(0.169)
Richer	**1.141+**	**1.484****	0.906	0.006	1.244
	(0.090)	(0.189)	(0.105)	(0.338)	(0.212)
Richest	**1.178+**	**1.968*****	1.145	**0.845***	1.038
	(0.108)	(0.314)	(0.162)	(0.374)	(0.191)
Semi-urban	0.868	0.758	1.028	-0.371	0.827
	(0.086)	(0.156)	(0.181)	(0.435)	(0.208)
Rural	**1.218****	0.848	0.886	0.183	**0.659***
	(0.089)	(0.119)	(0.103)	(0.297)	(0.110)
Male	**0.712*****	1.124	**0.503*****	**-0.653***	**0.693****
	(0.039)	(0.115)	(0.039)	(0.260)	(0.092)
Constant	3.200***	3.652***	9.090***	5.952***	9.575***
	(0.356)	(0.692)	(1.576)	(0.479)	(2.392)
Observations	10,193	10,331	10,121	7,771	7,765

Odds Ratios for logistic regressions and coefficients for OLS regressions; Standard errors in parentheses; Note: no education, never married, poorest, urban residence and female are controls for education, marital status, wealth status, residence type and gender dummies; lifetime sexual partners=total number of lifetime sexual partners; partners =total partners; l12M=Last 12 months; N=Number of observations; + p<.10, * p<.05, ** p<.01, *** p<.001

Multivariate Regression Model-Hearing Disability and HIV/AIDS Knowledge and Sexual Behaviour

	(1) Age first sex (OLS)	(2) last sex used condom	(3) genital sores l12M	(4) genital discharge l12M	(5) STD l12M	(6) can get condom	(7) number of partners l12M (OLS)	(8) total number of lifetime sexual partners (OLS)
Low Severity	-0.162	**1.805****	**1.635*****	**1.481***	**1.509****	0.920	**-0.273*****	0.386
	(0.163)	(0.345)	(0.242)	(0.228)	(0.231)	(0.125)	(0.079)	(0.419)
Age (years)	**0.036*****	**0.984****	0.998	0.998	**0.991***	**0.985*****	0.014	**0.073*****
	(0.004)	(0.006)	(0.004)	(0.004)	(0.004)	(0.003)	(0.009)	(0.009)
Primary Education	**0.951*****	**1.724*****	0.925	1.051	**1.034**	**1.320*****	**-0.288+**	0.016
	(0.072)	(0.149)	(0.068)	(0.080)	(0.077)	(0.076)	(0.117)	(0.166)
Secondary plus	**3.185*****	**1.708*****	**0.752***	**0.726***	0.830	**2.819*****	-0.079	-0.399
	(0.139)	(0.226)	(0.105)	(0.112)	(0.114)	(0.303)	(0.271)	(0.339)
Currently married	**0.576*****	**0.100*****	**3.265*****	**2.962*****	**4.630*****	**2.582*****	-0.052	**0.558****
	(0.100)	(0.010)	(0.351)	(0.338)	(0.545)	(0.185)	(0.159)	(0.176)
Formerly married	0.090	**0.597*****	**3.179*****	**3.191*****	**4.303*****	**2.848*****	0.514	**1.297*****
	(0.135)	(0.080)	(0.441)	(0.464)	(0.658)	(0.291)	(0.361)	(0.281)
Poorer	**-0.337*****	**1.320+**	**1.756*****	**1.735*****	**1.501****	**1.342****	0.185	**0.398****
	(0.102)	(0.218)	(0.202)	(0.221)	(0.201)	(0.125)	(0.172)	(0.152)
Middle	**-0.377*****	**1.726*****	**2.410*****	**2.803*****	**2.574*****	**1.409*****	-0.036	**0.544****
	(0.101)	(0.268)	(0.271)	(0.347)	(0.329)	(0.132)	(0.123)	(0.166)
Richer	**-0.592*****	**1.928*****	**2.391*****	**2.469*****	**2.572*****	**1.410*****	0.073	**0.911*****
	(0.107)	(0.291)	(0.272)	(0.313)	(0.334)	(0.128)	(0.155)	(0.195)
Richest	**-0.346****	**1.994*****	**2.105*****	**2.464*****	**2.493*****	1.154	0.247	**1.145*****
	(0.120)	(0.331)	(0.271)	(0.342)	(0.354)	(0.116)	(0.154)	(0.253)
Semi-urban	-0.358*	0.999	1.090	1.008	1.017	1.048	0.558	-0.178
	(0.140)	(0.146)	(0.161)	(0.149)	(0.143)	(0.124)	(0.436)	(0.269)
Rural	0.123	**0.765***	1.121	0.990	1.031	**0.710*****	-0.075	**-0.535***
	(0.100)	(0.087)	(0.108)	(0.097)	(0.100)	(0.055)	(0.123)	(0.234)
Male	**0.945*****	**1.704*****	**0.472*****	**0.360*****	**0.519*****	**6.481*****	0.092	**4.743*****
	(0.088)	(0.144)	(0.046)	(0.042)	(0.050)	(0.512)	(0.085)	(0.274)
Constant	14.625***	0.579**	0.030***	0.027***	0.026***	1.050	0.899***	-0.953*
	(0.161)	(0.120)	(0.005)	(0.005)	(0.005)	(0.133)	(0.255)	(0.390)
Observations	8,679	7,838	10,928	10,927	10,141	8,400	7,855	9,173

Odds Ratios for logistic regressions and coefficients for OLS regressions; Standard errors in parentheses; Note: no education, never married, poorest, urban residence and female are controls for education, marital status, wealth status, residence type and gender dummies; lifetime sexual partners=total number of lifetime sexual partners; partners =total partners; l12M=Last 12 months; N=Number of observations; + p<.10, * p<.05, ** p<.01, *** p<.001
