# Supplementary material for: HIV/AIDS knowledge, attitudes and behaviour of persons with and without disabilities from the Uganda Demographic and Health Survey 2011: Differential access to HIV/AIDS information and services
Source: PLoS One. 2017 Apr 13;12(4):e0174877. doi: 10.1371/journal.pone.0174877 (PMC5390986; doi:10.1371/journal.pone.0174877)
Supplement: S6 Table — (PDF) [file pone.0174877.s006.pdf]

Multivariate Logistic Model-Multiple Disability and HIV/AIDS Knowledge and awareness

	(2)<br>reduced risk HIV infection using condom	(3)<br>reduced risk HIV infection one partner	(4)<br>healthy looking person can have HIV	(5)<br>risk HIV infection mosquito bites	(6)<br>risk HIV infection share food	(7)<br>okay a teacher with HIV to teach	(8)<br>okay care for a relative with HIV	(9)<br>okay buy vegetables HIV infected vendor
Multiple disabilities	**1.315***	1.048	1.003	**1.314****	1.177	**0.705*****	1.280	**0.744****
	(0.182)	(0.188)	(0.155)	(0.133)	(0.146)	(0.070)	(0.206)	(0.075)
Age (years)	0.994	1.006	**1.010***	**0.991****	**0.982*****	**1.009****	**1.030*****	**1.015*****
	(0.004)	(0.005)	(0.005)	(0.003)	(0.004)	(0.003)	(0.005)	(0.003)
Primary Education	**1.136+**	1.128	**1.477*****	**0.526*****	**0.499*****	**2.301*****	**2.746*****	**2.334*****
	(0.076)	(0.103)	(0.125)	(0.029)	(0.035)	(0.137)	(0.260)	(0.132)
Secondary plus	**1.321***	1.178	**2.065*****	**0.229*****	**0.308*****	**6.170*****	**3.739*****	**5.381*****
	(0.163)	(0.208)	(0.390)	(0.0279)	(0.045)	(0.978)	(0.880)	(0.772)
Currently married	**1.346*****	1.141	**1.597*****	**1.135+**	1.080	**1.213****	**1.288****	1.113
	(0.111)	(0.125)	(0.148)	(0.076)	(0.088)	(0.085)	(0.122)	(0.075)
Formerly married	**1.483****	1.178	**2.033*****	1.131	1.146	**1.535*****	**1.605****	**1.277***
	(0.184)	(0.197)	(0.308)	(0.111)	(0.135)	(0.157)	(0.244)	(0.127)
Poorer	**1.175+**	1.202	**1.593*****	0.934	0.872	**1.312*****	**2.369*****	**1.413*****
	(0.112)	(0.144)	(0.159)	(0.070)	(0.078)	(0.098)	(0.227)	(0.102)
Middle	**1.518*****	**1.425****	**1.972*****	0.892	**0.774****	**1.504*****	**2.816*****	**1.510*****
	(0.152)	(0.178)	(0.212)	(0.069)	(0.073)	(0.117)	(0.290)	(0.113)
Richer	**1.737*****	**1.524****	**2.096*****	0.887	0.952	**1.544*****	**3.068*****	**1.577*****
	(0.175)	(0.195)	(0.219)	(0.069)	(0.087)	(0.122)	(0.326)	(0.118)
Richest	**1.660*****	**1.805*****	**3.319*****	**0.752****	**0.811+**	**1.944*****	**4.935*****	**1.820*****
	(0.184)	(0.272)	(0.481)	(0.0687)	(0.0874)	(0.185)	(0.713)	(0.164)
Semi-urban	1.022	1.443+	1.419	**1.466*****	1.050	**0.664****	1.100	**0.769***
	(0.148)	(0.314)	(0.308)	(0.168)	(0.146)	(0.084)	(0.236)	(0.089)
Rural	0.897	1.141	0.878	**1.242****	0.985	**0.726*****	0.887	**0.727*****
	(0.084)	(0.141)	(0.112)	(0.094)	(0.088)	(0.061)	(0.117)	(0.059)
Male	1.001	1.118	**1.795*****	1.053	**0.846***	0.964	1.029	**1.477*****
	(0.073)	(0.111)	(0.173)	(0.061)	(0.063)	(0.058)	(0.090)	(0.091)
Observations	9991	10565	10544	9538	10159	10533	10752	10822

Odds Ratios (except for OLS regressions, coefficients); Standard errors in parentheses; Note: no education, never married, poorest, urban residence and female are controls for education, marital status, wealth status, residence type and gender dummies; N=Number of observations; + p<.10, * p<.05, ** p<.01, *** p<.001

Multivariate Logistic Model-Multiple Disability and HIV/AIDS transmission

	(1)<br>HIV transmission possible during pregnancy	(2)<br>HIV transmission possible during delivery	(3)<br>HIV transmission possible during breastfeeding	(4)<br>Months since last HIV test (OLS)	(5)<br>Received last HIV test results
Multiple disabilities	1.133	0.880	0.942	**-0.900+**	**0.596****
	(0.118)	(0.156)	(0.152)	(0.462)	(0.116)
Age (years)	**0.989*****	**1.014***	0.999	**0.135*****	**1.025*****
	(0.003)	(0.006)	(0.005)	(0.013)	(0.007)
Primary Education	**0.743*****	**2.012*****	**1.253****	**-0.338**	**1.643*****
	(0.039)	(0.206)	(0.103)	(0.228)	(0.197)
Secondary-plus Education	**0.628*****	**4.252*****	**1.683*****	**-1.234*****	**3.023*****
	(0.054)	(1.093)	(0.263)	(0.346)	(0.804)
Currently Married	1.060	**2.053*****	**1.541*****	**0.506+**	1.132
	(0.067)	(0.234)	(0.152)	(0.275)	(0.166)
Formerly Married	1.122	**2.388*****	**1.517****	0.165	1.061
	(0.108)	(0.424)	(0.224)	(0.405)	(0.226)
Poorer	0.962	1.129	0.887	0.217	0.898
	(0.077)	(0.134)	(0.101)	(0.332)	(0.133)
Middle	1.100	**1.560*****	0.846	0.164	1.052
	(0.091)	(0.202)	(0.097)	(0.335)	(0.170)
Richer	**1.139+**	**1.467****	0.907	-0.003	1.269
	(0.090)	(0.188)	(0.106)	(0.338)	(0.218)
Richest	**1.179+**	**1.951*****	1.144	**0.839***	1.027
	(0.108)	(0.312)	(0.162)	(0.375)	(0.189)
Semi-Urban residence	0.868	0.761	1.032	-0.379	0.821
	(0.086)	(0.157)	(0.182)	(0.434)	(0.206)
Rural Residence	**1.218****	0.851	0.889	0.187	**0.646****
	(0.089)	(0.119)	(0.104)	(0.297)	(0.107)
Male	**0.712*****	1.121	**0.503*****	**-0.659***	**0.687****
	(0.039)	(0.114)	(0.039)	(0.260)	(0.091)
Constant				5.914***	
				(0.480)	
Observations	10182	10320	10112	7763	7757

Odds Ratios (except for OLS regressions, coefficients); Standard errors in parentheses; "Note: no education, never married, poorest, urban residence and female are controls for education, marital status, wealth status, residence type and gender dummies; N=Number of observations; Odds Ratios." + p<.10, * p<.05, ** p<.01, *** p<.001

Multivariate Regression Model-Multiple Disability and HIV/AIDS Knowledge and Sexual Behaviour

	(1) Age first sex (OLS)	(2) last sex used condom	(3) genital sores l12M	(4) genital discharge l12M	(5) STD l12M	(6) can get condom	(7) number of partners l12M (OLS)	(8) total number of lifetime sexual partners (OLS)
Multiple disabilities	-0.608***	1.598**	1.625***	1.729***	1.481**	0.915	-0.054	0.108
	(0.137)	(0.268)	(0.199)	(0.224)	(0.200)	(0.110)	(0. 257)	(0.282)
Age (years)	0.039***	0.983**	0.996	0.997	0.991*	0.985***	0.014	0.073***
	(0.004)	(0.00546)	(0.00386)	(0.00413)	(0.00404)	(0.00345)	(0.009)	(0.009)
Primary Education	0.944***	1.721***	0.927	1.054	1.033	1.318***	-0.226+	0.011
	(0.072)	(0.149)	(0.0686)	(0.0806)	(0.0773)	(0.0754)	(0.117)	(0.165)
Secondary plus	3.170***	1.715***	0.755*	0.735*	0.833	2.817***	-0.075	-0.406
	(0.139)	(0.226)	(0.105)	(0.113)	(0.114)	(0.303)	(0.271)	(0.340)
Currently married	0.559***	0.100***	3.290***	2.988***	4.639***	2.577***	-0.050	0.554**
	(0.100)	(0.0102)	(0.353)	(0.340)	(0.547)	(0.185)	(0.159)	(0.176)
Formerly married	0.093	0.596***	3.151***	3.158***	4.257***	2.853***	0.514	1.295***
	(0.134)	(0.0796)	(0.438)	(0.460)	(0.652)	(0.291)	(0.362)	(0.281)
Poorer	-0.327**	1.323+	1.748***	1.721***	1.499**	1.342**	0.182	0.398**
	(0.102)	(0.219)	(0.201)	(0.219)	(0.200)	(0.125)	(0.170)	(0.153)
Middle	-0.380***	1.729***	2.417***	2.808***	2.579***	1.408***	-0.039	0.550***
	(0.101)	(0.268)	(0.271)	(0.347)	(0.329)	(0.132)	(0.123)	(0.165)
Richer	-0.352***	1.930***	2.381***	2.462***	2.563***	1.412***	0.074	0.914***
	(0.120)	(0.291)	(0.270)	(0.311)	(0.332)	(0.128)	(0.155)	(0.916)
Richest	-0.353**	2.000***	2.117***	2.477***	2.497***	1.152	0.246	1.150***
	(0.140)	(0.331)	(0.272)	(0.343)	(0.355)	(0.116)	(0.154)	(0.252)
Semi-urban	-0.353*	1.003	1.087	1.008	1.019	1.050	0.554	-0.174
	(0.140)	(0.146)	(0.160)	(0.149)	(0.143)	(0.124)	(0.436)	(0.269)
Rural	0.128	0.765*	1.117	0.987	1.030	0.710***	-0.076	-0.534*
	(0.100)	(0.0862)	(0.107)	(0.0968)	(0.100)	(0.0552)	(0.123)	(0.234)
Male	0.942***	1.710***	0.473***	0.361***	0.519***	6.474***	0.092	4.745***
	(0.088)	(0.145)	(0.0464)	(0.0425)	(0.0504)	(0.512)	(0.085)	(0.274)
Constant	14.593						0.887	-0.954
	(0.161)						(0.256)	(0.393)
Observations	8673	7830	10917	10916	10130	8395	7847	9164

Odds Ratios (except for OLS regressions, coefficients); Standard errors in parentheses; Note: no education, never married, poorest, urban residence and female are controls for education, marital status, wealth status, residence type and gender dummies; N=Number of observations; l12M =Last 12 months. + p<.10, * p<.05, ** p<.01, *** p<.001
